# Reflections on an inflection: From virology to parasitology guided by POLARIS

**DOI:** 10.1371/journal.ppat.1006941

**Published:** 2018-06-14

**Authors:** Akhil B. Vaidya

**Affiliations:** Center for Molecular Parasitology, Drexel University College of Medicine, Philadelphia, Pennsylvania, United States of America; The Fox Chase Cancer Center, UNITED STATES

The lives of individuals are marked by inflection points, events that seem prosaic at the time of their occurrence but have profound consequences as life unfolds. For me, such an inflection point happened in 1980 while I was a virologist working on mouse mammary tumor virus: My colleague Bill Weidanz, a pioneering immunoparasitologist, suggested that we should construct a genomic library of a malaria parasite from which to fish out genes encoding protective antigens to produce a malaria vaccine. Being a somewhat cocky molecular virologist, I was quick to agree, saying it would be a “piece of cake.” Bill (whom we sadly lost last November) never missed a chance to rib me for that flippant comment! Developing a vaccine for malaria has been anything but a “piece of cake.” However, it was not the fishing for antigens but a quality control experiment to determine the robustness of our genomic library from *Plasmodium yoelii* that had profound personal consequences for me. We detected numerous phage clones in the library bearing repetitive DNA of this eukaryotic organism, thus showing that our library was of good quality. It was the study of this 6-kb repetitive DNA that altered the course of my scientific career (and led me to cancel my subscription to *Journal of Virology*).

We did not immediately know what this 6-kb repetitive DNA encoded but discovered that it was tandemly arranged head-to-tail in all species of malaria parasites and expressed as multiple RNA molecules of various sizes. At a molecular parasitology meeting in the mid-1980s, several investigators presented their exciting discoveries of important malaria parasite antigens that they named with clever acronyms, e.g., RESA (Ring-infected Erythrocyte Surface Antigen), FIRA (Falciparum Interspersed Repeat Antigen), and MESA (Mature parasite-infected Erythrocyte Surface Antigen). Not to be outdone, I coined an acronym for our repetitive DNA: POLARIS (Plasmodium-Origin Large Array of Relatively Invariant Sequence). Although I thought the acronym was clever, if not funny, the editor of the journal *Molecular Parasitology* was not amused and struck it out of the paper we published in 1987. POLARIS thus remains an obscure insider joke.

Nevertheless, like its celestial namesake, POLARIS has guided the research in my laboratory for 30 years. When we completed the sequence of the 6-kb DNA molecule in 1988, it revealed itself to be the mitochondrial DNA (mtDNA) of malaria parasites. At that time, some researchers were already investigating a 35-kb circular DNA molecule that they believed to be the parasite’s mtDNA. However, based on our sequence data establishing the 6-kb tandemly repeated DNA as the mitochondrial genome, it became clear that the 35-kb circular DNA molecule was something else. Upon further sequencing, the 35-kb DNA molecule was found to be a highly reduced chloroplast DNA, which was subsequently shown to reside in a separate cytoplasmic organelle now known as the apicoplast. Not just malaria parasites but all apicomplexan parasites were thus revealed to have originated from an algal progenitor! These insights have significant implications for discovering control measures for apicomplexan parasites, given that they display certain plant-like physiological processes that can be targeted for selective inhibition.

The malaria parasite mtDNA turns out to be the smallest known among eukaryotes, encoding just 3 proteins and scrambled pieces of ribosomal RNA. Over the years, my colleagues and I noted significant differences in the active sites of the mitochondrial electron transport chain (mtETC) protein cytochrome *b*, which we showed to be the reason for the selective toxicity of certain antimalarial drugs. Selective inhibition of parasite mtETC has proven to be a fertile area for antimalarial drug discovery. Our demonstration that the parasite mtDNA is inherited from female gametes has allowed determination of directionality of mating in the progeny of parasite genetic crosses; in one cross, all progeny had the same maternal parent, suggesting unidirectional incompatibility, the significance of which remains to be explored. We also found that malarial mtDNA is highly conserved across distant parasite isolates, which has allowed others to establish phylogenetic relationships among parasite isolates. We have carried out extensive genetic studies to understand the importance of various canonical pathways usually relegated to the mitochondrion. It seems the only essential function of the mtETC in blood stages of *P*. *falciparum* is to provide oxidized ubiquinone, serving mitochondrially located pyrimidine biosynthesis enzyme dihydroorotate dehydrogenase (DHOD). Right now, there are clinical trials underway with parasite DHOD inhibitors being developed as antimalarial drugs. We also found that most of the tricarboxylic acid cycle enzymes are dispensable in the blood stages of *P*. *falciparum* but become critical in insect stages. Overall, the picture of the parasite mitochondrion that emerges from all the studies thus far is that of an organelle with minimal but very essential functions for parasite survival and thus a very attractive target for antimalarial drugs.

My metamorphosis into a parasitologist has been immensely rewarding. I have derived great pleasure in knowing and working with outstanding colleagues all over the world. Recently, we discovered yet another pathway in malaria parasites that is inhibited by a large number of chemical classes with antimalarial properties, some of which are undergoing clinical trials. I remain very excited to explore the intricacies of this pathway. It would be wonderful to see one of these compounds deployed as an antimalarial drug that could potentially save millions of lives. I have never regretted canceling my *Journal of Virology* subscription.

**Image 1:**
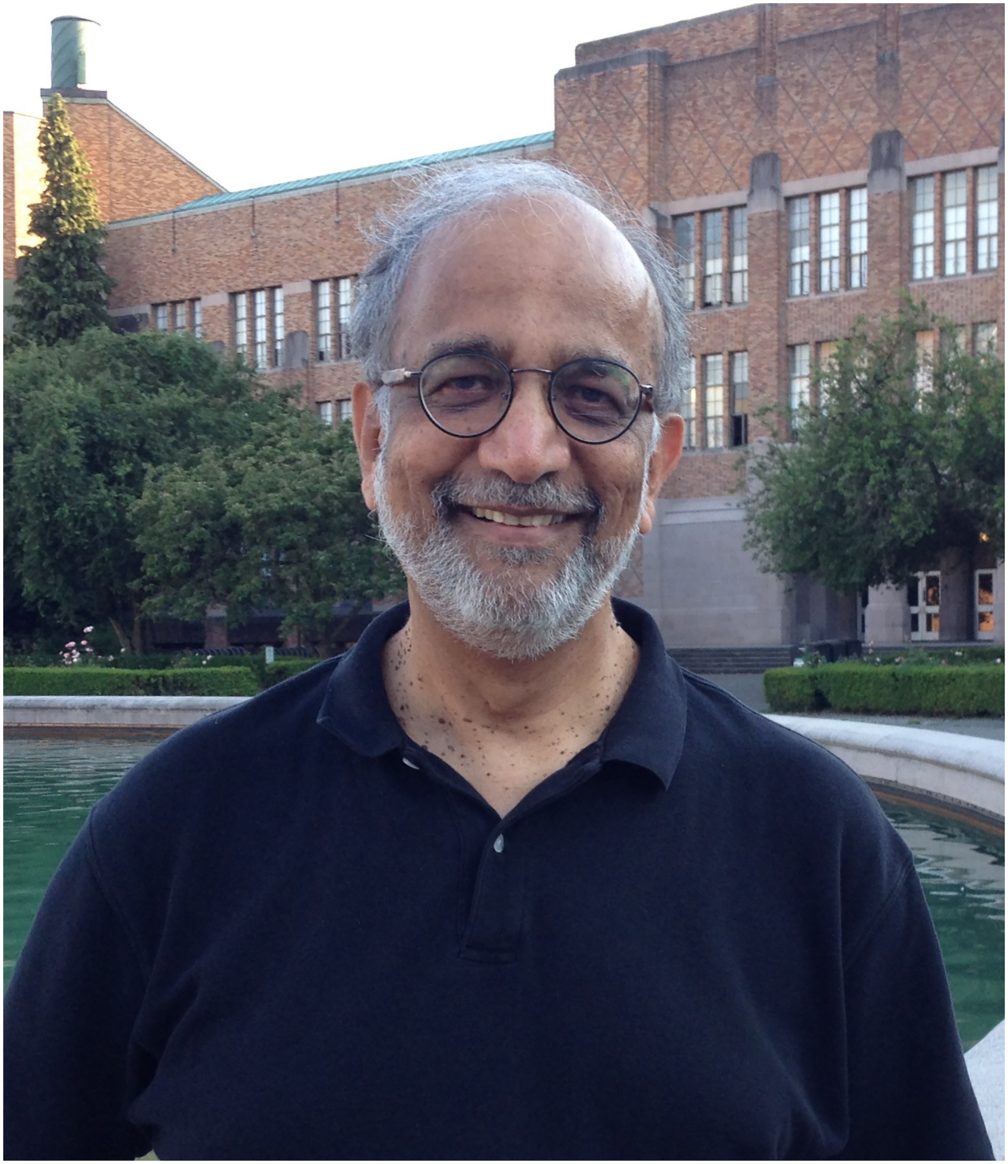
Akhil B. Vaidya.

